# Yakuchinone B Ameliorates DSS-Induced Colitis by Modulating the Gut Microbiota-Metabolite Axis

**DOI:** 10.3390/nu18162628

**Published:** 2026-08-12

**Authors:** Yang Wang, Wang Peng, Wei Fan, Hang Xiao, Shiyin Guo, Zhonghai Tang, Jingping Qin

**Affiliations:** 1 College of Food Science and Technology, Hunan Agricultural University, Changsha 410128, China; 2 Hunan Engineering Research Center for Nutrition, Health and Deep Processing of Rapeseed Oil, Hunan Agricultural University, Changsha 410128, China; 3 Department of Food Science, University of Massachusetts, Amherst, MA 01003, USA; 4 College of Bioscience and Biotechnology, Hunan Agricultural University, Changsha 410128, China

**Keywords:** Yakuchinone B, DSS-induced colitis, gut microbiota, anti-inflammation, metabolomics, short-chain fatty acids

## Abstract

**Background/Objectives:** Inflammatory bowel disease (IBD) is a chronic and recurrent gastrointestinal disorder characterized by intestinal inflammation and gut microbiota dysbiosis, but current therapies remain limited by adverse effects and suboptimal long-term efficacy. **Methods:** Here, using a dextran sulfate sodium (DSS)-induced mouse model of IBD-like colitis, we investigated the protective effects of Yakuchinone B (YB)—a diarylheptanoid derived from *Alpinia oxyphylla* with reported anti-inflammatory and antioxidant activities—against inflammatory bowel disease (IBD). **Results:** YB supplementation significantly alleviated colitis symptoms, as evidenced by reduced body weight loss, lower disease activity index scores, attenuated colonic shortening, and ameliorated histopathological damage. YB also decreased the colonic and serum levels of TNF-α, IL-1β, and IL-6. Microbiome profiling showed that YB restored gut microbial diversity and reshaped microbial composition, with increased abundances of *Alistipes* and *Duncaniella* and reduced overgrowth of *Akkermansia*. Untargeted metabolomics revealed that YB modulated colitis-associated pathways, including purine metabolism, alanine, aspartate, and glutamate metabolism, and steroid hormone biosynthesis. Targeted analysis further showed that YB increased acetate, propionate, and butyrate levels. **Conclusions:** These results collectively suggest that YB ameliorates DSS-induced colitis by attenuating inflammation, associated with modulation of the gut microbiota-host metabolism axis, and promoting short-chain fatty acid production, supporting its potential as a promising functional dietary candidate for IBD management.

## 1. Introduction

Inflammatory bowel disease (IBD) is a chronic and relapsing gastrointestinal disorder characterized by persistent inflammation and ulceration of the intestinal mucosa, primarily affecting the small intestine and/or colon [1]. Crohn’s Disease (CD) and Ulcerative Colitis (UC) are the two major clinical forms of IBD, and their incidence has continued to increase worldwide, imposing a significant public health burden [2]. Although the precise etiology of IBD remains incompletely understood, its pathogenesis is generally considered to involve a complex interplay among genetic susceptibility, epithelial barrier dysfunction, dysregulated immune responses, gut microbiota dysbiosis, and environmental factors [3].

Current conventional therapies for IBD, including aminosalicylates (e.g., sulfasalazine), corticosteroids, immunosuppressants (e.g., azathioprine), and biologics, are often hampered by adverse effects, loss of response, and high relapse rates following long-term use [4,5]. Therefore, the discovery of safe, effective, and sustainable alternative interventions is of paramount importance. Recently, food-derived natural products, such as polysaccharides [6,7], plant polyphenols [8,9], and plant pigments [10], have attracted increasing attention as potential dietary interventions for IBD. A growing body of evidence highlights that the gut microbiota plays a pivotal role in modulating intestinal immunity, and the targeted restoration of microbial homeostasis has emerged as a potentially effective strategy for alleviating IBD symptoms [11,12]. Crucially, many phytochemicals exert their protective effects by reshaping the gut microbial architecture [13]. For instance, curcumin ameliorates murine colitis by enriching beneficial taxa such as Muribaculaceae and *Muribaculum*, functioning as a potent prebiotic-like agent [14]. Similarly, dietary supplementation with resveratrol attenuates inflammation-associated intestinal injury in colitis models by restricting the overgrowth of pathobionts (e.g., *Akkermansia*, *Dorea*, *Sutterella*, and *Bilophila*) while promoting *Bifidobacterium* populations [15].

Yakuchinone B (YB, 1-[4′-hydroxy-3′-methoxy-phenyl]-7-phenylhept-1-en-3-one) is a prominent bioactive diarylheptanoid isolated from *Alpinia oxyphylla*. Previous studies have shown that YB and its derivatives can inhibit Islet amyloid polypeptide (IAPP) aggregation and may therefore have therapeutic potential in Type 2 diabetes mellitus [16]. In addition, 6-Gingerol, Yakuchinone A, and YB have been identified by computational analyses as putative inhibitors of COX-2, IKK, and TBK-1, respectively [17]. However, the in vivo biological activity of YB against colitis remains unknown. In the present study, we investigated whether YB can ameliorate DSS-induced colitis and explored the potential mechanisms underlying its effects, with particular emphasis on the inflammatory response, gut microbiota remodeling, and metabolite alterations.

## 2. Materials and Methods

### 2.1. Materials

Yakuchinone B (CAS: 81840-57-5, purity > 95%) was supplied by Jinan Mingke Biotechnology Co., Ltd. (Jinan, China). Colitis-inducing DSS (36–50 kDa) was procured from MP Biomedicals (Irvine, CA, USA). ELISA kits for TNF-α, IL-6, and IL-1β were provided by Nanjing Jiancheng Bioengineering Institute (Nanjing, China). Paraffin, neutral balsam, hematoxylin, and eosin were purchased from Changsha Aibiwei Biotechnology Co., Ltd. (Changsha, China). Agarose (Biowest, Nuaillé, France, Cat. No. 800007), Buffer EB (250 mL, QIAGEN, Hilden, Germany, Cat. No. 1014609), and VAHTS HiFi Universal Amplification Mix (Vazyme Biotech Co., Ltd., Nanjing, China, Cat. No. Q111-02) were also used in this study.

### 2.2. Animal Experiment

All animal experiments were conducted in accordance with institutional guidelines and were approved by the Animal Committee of South China Agricultural University (protocol number: 2023B097). Fifty male CD-1 mice were purchased from Charles River Laboratories (Beijing, China). The mice were housed under controlled conditions (25 ± 2 °C, 65% relative humidity, and a 12-h light/dark cycle) with *ad libitum* access to food and water. During the initial 1-week acclimation period, all mice were fed a standard AIN-93G diet, and cages were systematically rotated to minimize baseline variations in gut microbiota.

**Figure 1:**
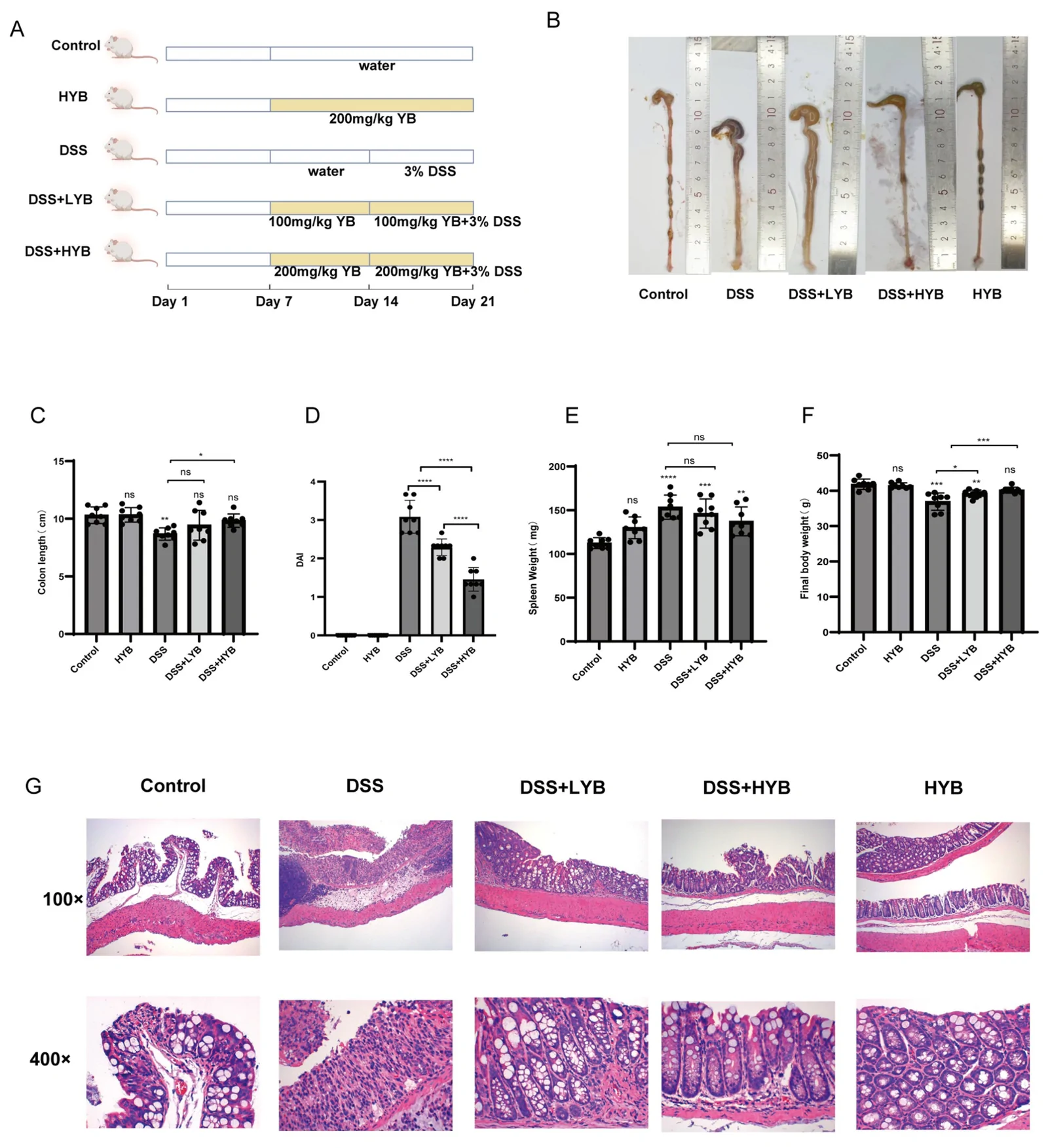
YB alleviates DSS-induced colitis symptoms in mice. (**A**) Schematic illustration of experimental design. (**B**) Representative colon images. (**C**) Colon length. (**D**) Disease activity index (DAI) scores. (**E**) Spleen weight. (**F**) Final body weight. (**G**) Representative images of H&E-stained colonic sections (magnification: 100× and 400×). The yellow color in Figure A indicates the treatment phase. Data are presented as the mean ± SEM (*n* = 8 per group). * *p* < 0.05, ** *p* < 0.01, *** *p* < 0.001, and **** *p* < 0.0001. ns indicates not significant.

Following acclimation, the mice were randomly allocated into five groups (*n* = 10 per group): Control, DSS, DSS + LYB (low-dose YB, 100 mg/kg), DSS + HYB (high-dose YB, 200 mg/kg), and HYB (Figure 1A). Yakuchinone B (YB) was dissolved in a 0.5% sodium carboxymethyl cellulose (CMC-Na) vehicle solution. During the 7-day pre-treatment period (Days 8–14), mice in the DSS + LYB group were intragastrically administered 100 mg/kg YB daily, while the DSS + HYB and HYB groups received 200 mg/kg YB. Mice in the Control and DSS groups were administered equal volumes of the 0.5% CMC-Na vehicle. To induce experimental colitis, mice in the DSS, DSS + LYB, and DSS + HYB groups were provided with drinking water containing 3% (*w*/*v*) DSS from Days 14 to 20 [9,18]. Ten mice per group were enrolled. During the experiment, animals with bite wounds from cage aggression or severe DSS-induced colitis (weight loss > 20%, severe diarrhea, or rectal prolapse) were excluded.

Disease activity parameters, including body weight, stool consistency, and the presence of fecal blood, were monitored daily throughout the experiment. On Day 21, all mice were euthanized. Spleens were promptly excised and weighed to assess splenomegaly. Colonic contents were collected and immediately stored at −80 °C for subsequent gut microbiota and metabolomic analyses. Concurrently, colon tissues were excised, measured, and fixed in 4% neutral buffered formalin for downstream histological evaluation.

### 2.3. Animal Experimental Phenotypic Analysis

The disease activity index (DAI) was evaluated based on three clinical parameters: body weight loss, stool consistency, and fecal occult blood. The scoring system was defined as follows: body weight loss (0, no loss; 1, 1–5% loss; 2, 5–10% loss; 3, 10–20% loss), stool consistency (0, normal; 1, soft but formed; 2, very soft; 3, diarrhea), and fecal blood (0, no visible blood; 1, trace blood in some stool pellets; 2, blood regularly observed in stool; 3, gross bleeding in all stool pellets). For histopathological evaluation, colonic tissues were fixed in 4% neutral buffered formalin, embedded in paraffin, sectioned, and stained with hematoxylin and eosin (H&E). Representative images were captured using an Olympus BX53 optical microscope (Olympus, Tokyo, Japan) at 100× and 400× magnifications.

### 2.4. Measurement of Inflammatory Cytokines in Colonic Tissue

Approximately 30 mg of colon tissue was homogenized in ice-cold phosphate-buffered saline (PBS) at a ratio of 1:10 (*w*/*v*). The tissue homogenates were then centrifuged at 5000× *g* for 5 min at 4 °C. The resulting supernatants were carefully collected and either used immediately or aliquoted and stored at −80 °C until further analysis. Whole blood was collected and allowed to clot at room temperature for 30 min. After centrifugation at 1500–2000× *g* for 10–15 min, the supernatant was carefully transferred for subsequent analysis. The concentrations of interleukin-1 beta (IL-1β), interleukin-6 (IL-6), and tumor necrosis factor-alpha (TNF-α) in the colonic supernatants and serum were quantified using commercial enzyme-linked immunosorbent assay (ELISA) kits, strictly following the manufacturer’s instructions.

### 2.5. Quantification of Short-Chain Fatty Acids (SCFAs)

For the targeted analysis of SCFAs, approximately 25 mg of cecal contents from each mouse was suspended in 500 μL of 0.5% phosphoric acid. The mixture was vortexed for 5 min and subsequently centrifuged at 18,000× *g* for 15 min at 4 °C. The resulting supernatant was then extracted with 500 μL of diethyl ether by vigorous vortexing for 1 min, followed by a second centrifugation step at 18,000× *g* for 15 min at 4 °C. Finally, the upper organic phase was carefully collected, filtered, and analyzed using a gas chromatography–mass spectrometry (GC-MS) system (Agilent Technologies, Santa Clara, CA, USA).

### 2.6. Gut Microbiota Analysis by 16S rRNA Gene Sequencing

Total microbial genomic DNA was extracted from colonic contents using a commercial DNA extraction kit (Qiagen, Shanghai, China) in strict accordance with the manufacturer’s protocol. The concentration and purity of the extracted DNA were evaluated using a NanoDrop spectrophotometer (Thermo Fisher Scientific, Waltham, MA, USA), and DNA integrity was verified by agarose gel electrophoresis. The purified DNA served as the template for PCR amplification of the 16S rRNA gene with barcoded primers and Tks Gflex DNA polymerase. The resulting PCR amplicons were verified by gel electrophoresis, purified, precisely quantified using a Qubit dsDNA Assay Kit (Thermo Fisher Scientific, Waltham, MA, USA), and then sequenced.

For downstream bioinformatics analysis, raw sequencing data were processed and clustered into operational taxonomic units (OTUs) at a 97% sequence similarity threshold using the VSEARCH algorithm. Representative sequences for each OTU were extracted and taxonomically annotated using the QIIME pipeline https://qiime2.org/.

### 2.7. Fecal Untargeted Metabolomics Analysis

For the untargeted fecal metabolomics analysis, approximately 50 mg of each fecal sample was mixed with 800 μL of a pre-cooled extraction solvent consisting of methanol, acetonitrile, and water (MeOH:ACN:H_2_O, 2:2:1, *v*/*v*/*v*). The mixture was vortexed for 1 min and sonicated in an ice-water bath for 10 min, followed by centrifugation at 12,000 rpm for 15 min at 4 °C. The resulting supernatant was filtered and transferred to an autosampler vial for LC-MS/MS analysis. To monitor instrument stability, quality control (QC) samples were prepared by pooling equal aliquots (10 μL) from each processed sample.

LC-MS/MS analysis was performed using an Agilent 6540 Q-TOF mass spectrometer (Agilent Technologies, Santa Clara, CA, USA). Chromatographic separation was conducted on an Agilent Poroshell 120 PFP column (2.7 μm) at 40 °C, with a 5 μL injection volume. The flow rate was set at 0.3 mL/min. The mobile phase comprised (A) ultrapure water and (B) 0.1% formic acid in acetonitrile. The gradient program was: 0–15 min, 5–40% B; 15.01–20 min, 40.01–60% B; 20.01–30 min, 60.01–95% B; and 30.01–40 min, 5% B for re-equilibration. The same gradient was applied for both positive and negative ion modes. Mass spectrometry was performed on an ESI source operated in AutoMS mode. The drying gas (300 °C, 8 L/min) and sheath gas (350 °C, 11 L/min) were used for desolvation. The ion spray voltage was set at 4500 V, with a fragmentor voltage of 175 V. Full-scan MS and MS/MS spectra were acquired over the range of m/z 50–1050, with MS/MS triggered for ions with an intensity > 1000 counts. Collision energy was applied in a stepped cycle of 15, 30, and 45 eV. Raw data were collected over a 40-min acquisition period.

Raw data files were converted into the Analysis Base File (ABF) format and subsequently processed using the open-source software MS-DIAL (version 4.24). According to the Metabolomics Standards Initiative (MSI) criteria, metabolite annotations were assigned as level 2 confidence because authentic standards were not used for confirmation. The aligned dataset was then imported into the MetaboAnalyst 5.0 web platform for multivariate statistical analysis. Principal component analysis (PCA), partial least-squares discriminant analysis (PLS-DA), and orthogonal partial least-squares discriminant analysis (OPLS-DA) were employed to characterize overall metabolic variations and identify significantly altered metabolites between groups. Metabolites meeting the criteria of variable importance in the projection (VIP) > 1, fold change (FC) > 1.5 or <0.75, and *p* < 0.05 were considered significantly differential. Finally, pathway enrichment analysis was conducted against the Kyoto Encyclopedia of Genes and Genomes (KEGG) database to elucidate the biological relevance of these differential metabolites.

### 2.8. Data Analysis

All data are presented as the mean ± SEM. Statistical analyses were conducted using GraphPad Prism 11.0. Differences among multiple groups were analyzed by one-way analysis of variance (ANOVA) followed by Tukey’s post hoc test. Comparisons among multiple groups were evaluated using a one-way analysis of variance (ANOVA) followed by Tukey’s post hoc test. For comparisons between two independent groups, an unpaired Student’s *t*-test or a Wilcoxon rank-sum test was employed, depending on the data distribution. Statistical significance was defined as *p* < 0.05. Spearman’s correlation analyses and subsequent heatmap visualizations were generated using the OECloud platform (OE Biotech, Shanghai, China).

## 3. Results

### 3.1. YB Alleviates DSS-Induced Colitic Symptoms in Mice

To evaluate the protective effect of YB against colitis, mice were pretreated with water, low-dose YB (LYB, 100 mg/kg) or high-dose YB (HYB, 200 mg/kg) before administration of 3% DSS to induce experimental colitis (Figure 1A). No significant differences in gross appearance, behavior, body weight, spleen weight, or water intake were observed between the Control and HYB groups, confirming that YB caused no overt systemic toxicity under the experimental conditions. DSS treatment induced pronounced clinical manifestations of colitis, including pronounced colon shortening, increased disease activity index (DAI) scores, splenomegaly, and body weight loss (Figure 1B–G). In contrast, both LYB and HYB administration effectively reversed colon shortening (Figure 1B,C); YB treatment reduced DAI scores in a dose-dependent manner, with the HYB group achieving a notable 50.57% reduction compared to the DSS group (Figure 1D), and prevented spleen enlargement (Figure 1E). Furthermore, it attenuated body weight loss (Figure 1F). Histological analysis further confirmed the protective effect of YB against DSS-induced colonic injury. H&E staining showed that DSS treatment caused severe mucosal damage, characterized by epithelial disruption, crypt distortion, goblet cell loss, and inflammatory cell infiltration, whereas YB treatment markedly alleviated these pathological changes and partially restored mucosal architecture (Figure 1G). Collectively, these results demonstrate that oral administration of YB effectively alleviates DSS-induced colitis in mice without causing obvious adverse effects.

### 3.2. YB Attenuates DSS-Induced Colonic and Serum Inflammation by Suppressing Pro-Inflammatory Cytokine Production

**Figure 2:**
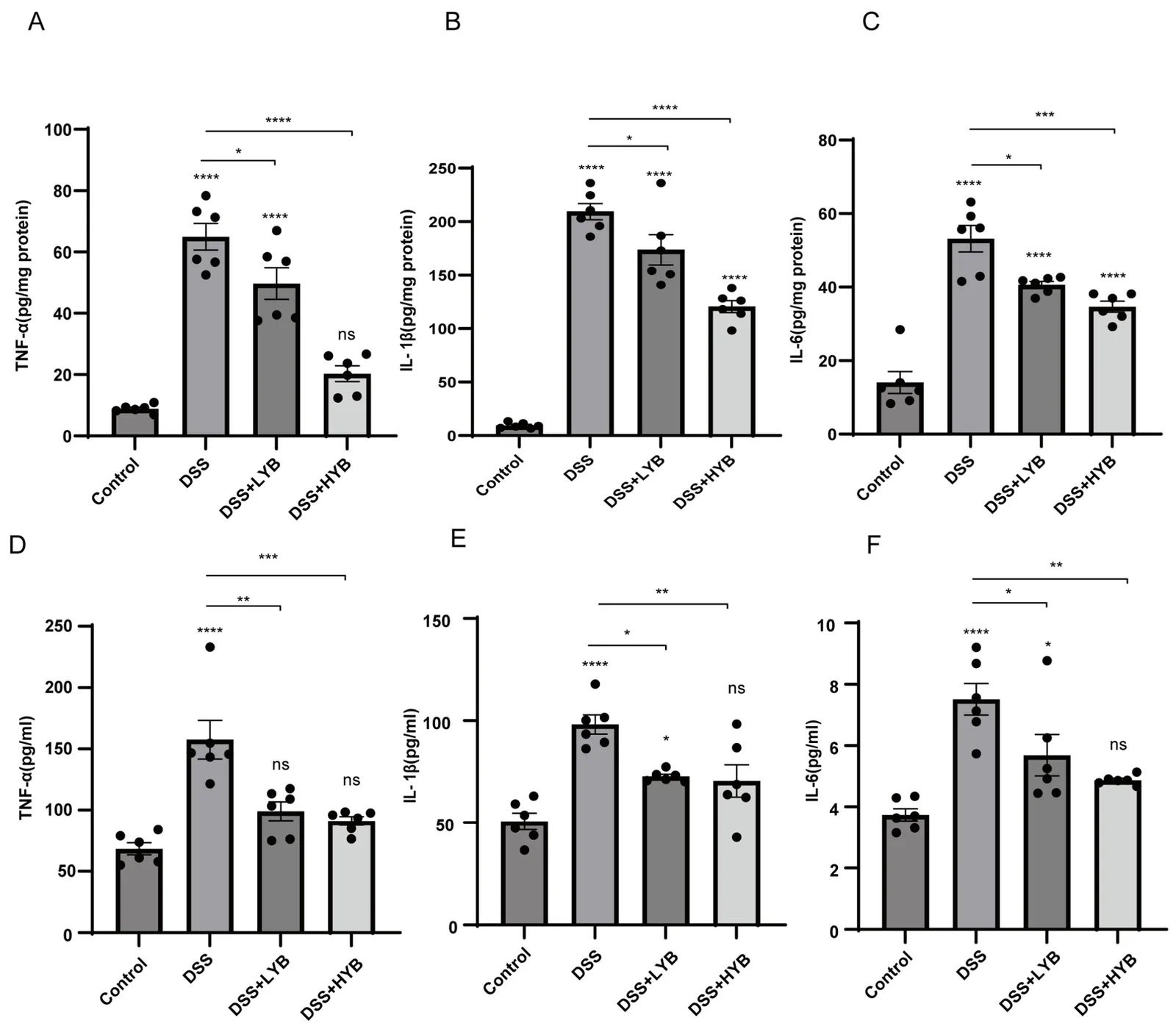
YB suppresses DSS-induced pro-inflammatory cytokine production in colonic tissues and serum. The levels of (**A**) TNF-α, (**B**) IL-1β, and (**C**) IL-6 in colonic tissues were measured by ELISA. The levels of (**D**) TNF-α, (**E**) IL-1β, and (**F**) IL-6 in serum were measured by ELISA. Data are presented as the mean ± SEM (*n* = 6 per group). * *p* < 0.05, ** *p* < 0.01, *** *p* < 0.001, and **** *p* < 0.0001. ns indicates not significant.

Excessive production of pro-inflammatory cytokines is a key feature of colitis and contributes to intestinal inflammatory injury [19]. Disruption of the intestinal barrier can trigger inflammatory cascades, leading to increased production of cytokines such as TNF-α, IL-1β, and IL-6 [20,21]. As shown in Figure 2A–C, DSS treatment markedly increased the colonic levels of TNF-α, IL-1β, and IL-6 to 7.32-, 21.66-, and 3.79-fold of the Control levels, respectively. In contrast, YB administration significantly reduced DSS-induced cytokine overproduction. LYB and HYB decreased TNF-α levels by 23.53% and 68.79%, IL-1β levels by 24.97% and 34.79%, and IL-6 levels by 17.10% and 42.48%, respectively, compared with the DSS group. Meanwhile, DSS treatment markedly increased the serum levels of TNF-α, IL-1β, and IL-6 to 4.03-, 1.93-, and 2.01-fold of the Control levels. LYB and HYB also decreased TNF-α levels by 37.10% and 42.17%, IL-1β levels by 25.86% and 28.15%, and IL-6 levels by 24.35% and 35.13% (Figure 2D–F). These findings suggest that YB alleviates DSS-induced colonic inflammation, at least in part, by suppressing the production of TNF-α, IL-1β, and IL-6.

### 3.3. YB Modulates Gut Microbiota Diversity and Composition in DSS-Induced Colitic Mice

**Figure 3:**
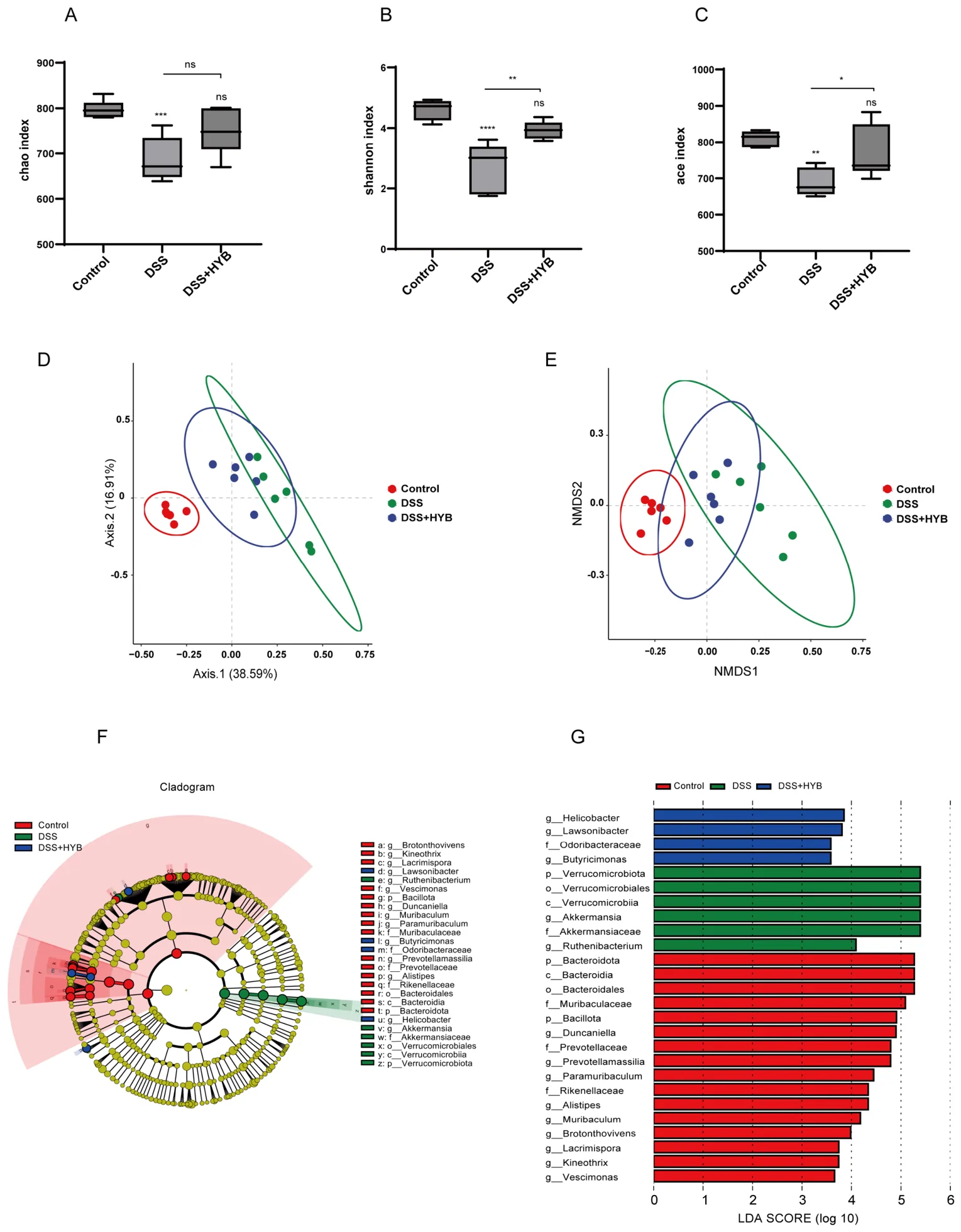
YB modulates gut microbial diversity and community structure in DSS-induced colitic mice. (**A**) Chao index, (**B**) Shannon index, and (**C**) ACE index evaluating the α-diversity of the gut microbiota. (**D**) Principal coordinate analysis (PCoA) and (**E**) non-metric multidimensional scaling (NMDS) plots illustrating the β-diversity among the different groups. (**F**) Taxonomic cladogram from LEfSe analysis depicting the phylogenetic distribution of gut microbiota. (**G**) Linear discriminant analysis (LDA) scores identifying the differentially abundant bacterial taxa among the groups. Data are presented as the mean ± SEM (*n* = 6 per group). * *p* < 0.05, ** *p* < 0.01, *** *p* < 0.001, and **** *p* < 0.0001. ns indicates not significant.

Based on its superior protective efficacy and absence of overt toxicity, the HYB group was selected for subsequent analyses. Given the close association between gut microbiota dysbiosis and IBD [22], 16S rRNA gene sequencing was performed to evaluate the effect of YB on gut microbial composition in DSS-induced colitic mice. As shown in Figure 3A–C, DSS treatment significantly reduced microbial richness and diversity, as indicated by decreased Chao, Shannon, and ACE indices. In contrast, HYB partially restored these DSS-induced reductions. Beta-diversity analysis based on Bray–Curtis distances, visualized by principal coordinates analysis (PCoA) and non-metric multidimensional scaling (NMDS), showed a clear separation between the DSS and Control groups (Figure 3D,E), indicating marked disruption of gut microbial structure after DSS treatment. YB supplementation reversed these structural shifts and clustered the microbiota composition closely toward that of the Control group, suggesting partial restoration of gut microbiota composition.

LEfSe analysis further identified distinct microbial signatures among groups. The Control group was predominantly enriched with commensal taxa, including *Duncaniella, Prevotellamassilia*, *Paramuribaculum*, *Alistipes*, *Muribaculum*, *Lacrimispora*, *Kineothrix*, and *Vescimonas*, whereas DSS induction triggered a pathogenic overgrowth of *Akkermansia* and *Ruthenibacterium.* Notably, YB intervention significantly reshaped the microbial profile, uniquely enriching *Helicobacter*, *Lawsonibacter*, and *Butyricimonas* (Figure 3F,G). Overall, these findings indicate that YB partially restores microbial diversity and reshapes gut microbial community structure in DSS-induced colitic mice, although the specific enrichment of taxa like *Helicobacter* warrants cautious interpretation regarding its context-dependent roles.

**Figure 4:**
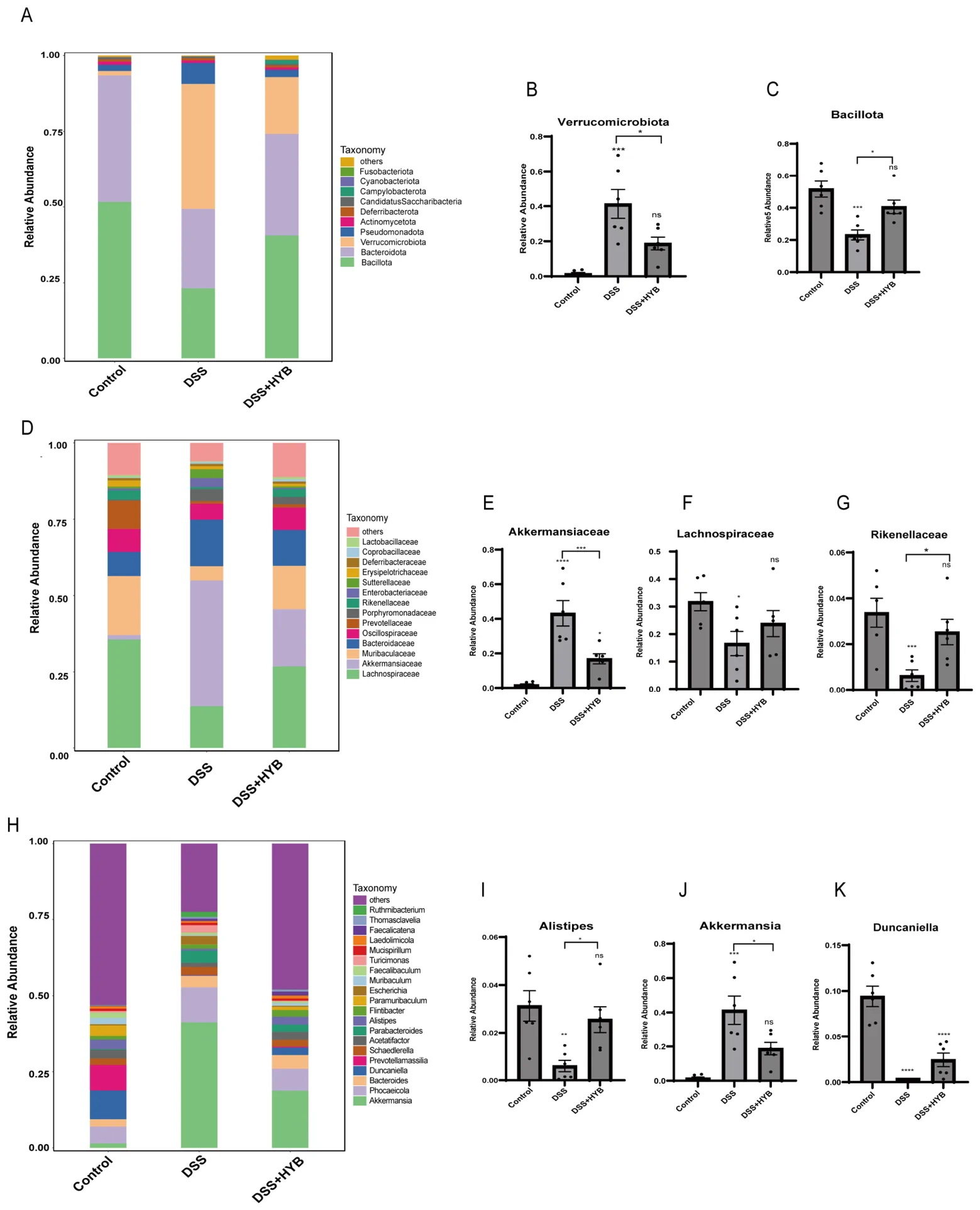
YB reshapes gut microbial composition at different taxonomic levels in DSS-induced colitic mice. (**A**) Taxonomic composition of the gut microbiota at the phylum level, and the relative abundances of (**B**) *Verrucomicrobiota* and (**C**) *Bacillota*. (**D**) Taxonomic composition at the family level, and the relative abundances of (**E**) *Lachnospiraceae*, (**F**) *Akkermansiaceae*, and (**G**) *Rikenellaceae*. (**H**) Taxonomic composition at the genus level, and the relative abundances of (**I**) *Alistipes,* (**J**) *Akkermansia,* and (**K**) *Duncaniella.* Data are presented as the mean ± SEM (*n* = 6 per group). * *p* < 0.05, ** *p* < 0.01, *** *p* < 0.001, and **** *p* < 0.0001. ns indicates not significant.

We next assessed microbiota composition at the phylum, family, and genus levels to further characterize the specific taxonomic shifts driven by YB. At the phylum level, DSS treatment markedly increased the relative abundance of *Verrucomicrobiota* and decreased that of *Bacillota*, consistent with previous reports showing reduced *Bacillota*-related taxa and enriched Verrucomicrobiota in colitis or IBD [23,24]. YB supplementation restored *Bacillota* to a level comparable to that of the Control group and markedly reduced *Verrucomicrobiota* (Figure 4A–C).

At the family level, the gut microbiota was mainly composed of *Lachnospiraceae*, *Akkermansiaceae*, *Muribaculaceae*, *Bacteroidaceae*, *Oscillospiraceae*, *Prevotellaceae*, *Rikenellaceae*, and *Porphyromonadaceae* (Figure 4D). DSS induced an abnormal expansion of *Akkermansiaceae*, *Lachnospiraceae*, and *Rikenellaceae*, which was notably attenuated by YB (Figure 4E–G). Given the diverse, context-dependent functions of *Lachnospiraceae* and *Rikenellaceae* members [25,26], these corrections highlight a complex ecological remodeling.

At the genus level, YB intervention completely corrected the DSS-induced overgrowth of *Akkermansia*—the primary driver of the Akkermansiaceae expansion—while robustly rescuing the severely depleted abundances of *Alistipes* and *Duncaniella* (Figure 4H–K). Although *Akkermansia* is generally considered a beneficial mucin-degrading bacterium, its bloom during DSS-induced colitis likely reflects excessive mucus utilization secondary to severe mucosal damage [27,28,29]. Conversely, the significant enrichment of *Alistipes* and *Duncaniella* by YB aligns with their well-documented protective roles against intestinal inflammation [30,31]. Taken together, these findings suggest that YB alleviates DSS-induced colitis by correcting taxonomic dysbiosis and enriching protective bacterial consortia.

### 3.4. YB Restores Short-Chain Fatty Acid Production and Reshapes the Gut Metabolome in Colitic Mice

**Figure 5:**
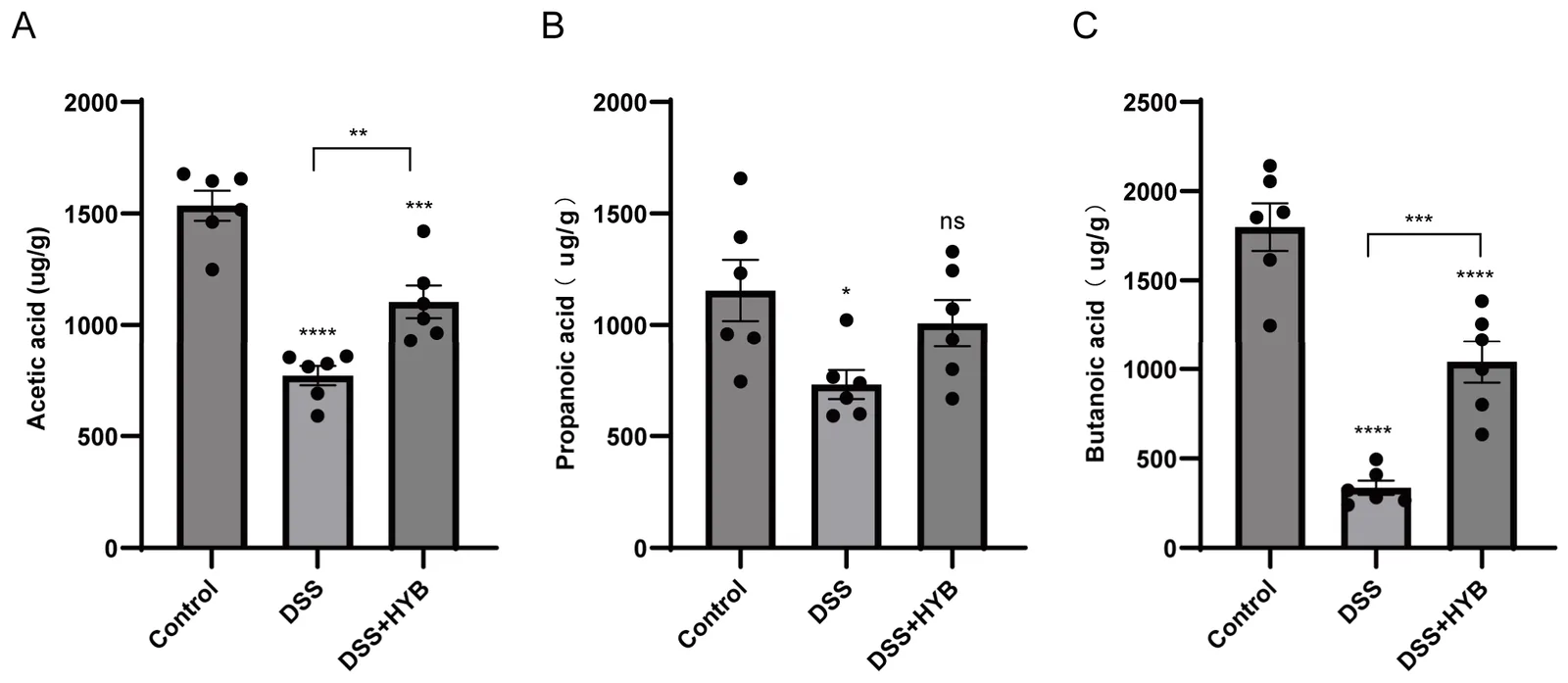
HYB restores short-chain fatty acid (SCFA) production in the cecum of DSS-induced colitic mice. Concentrations of (**A**) acetic acid, (**B**) propionic acid, and (**C**) butyric acid in the cecal contents. Data are presented as the mean ± SEM (*n* = 6 per group). * *p* < 0.05, ** *p* < 0.01, *** *p* < 0.001, and **** *p* < 0.0001. ns indicates not significant.

Short-chain fatty acids (SCFAs), notably acetic, propionic, and butyric acids, are crucial for reinforcing gut barrier integrity, supporting epithelial metabolism, and suppressing mucosal inflammation [32]. As shown in Figure 5A–C, DSS exposure significantly reduced cecal SCFA concentrations compared with the Control group. Strikingly, HYB robustly restored the production of these critical SCFAs. These results indicate that the protective effect of YB may be associated with enhanced SCFA production.

Beyond SCFA depletion, broader metabolic disturbances hallmark IBD pathogenesis. Thus, we profiled fecal metabolite alterations using an untargeted metabolomics approach.

**Figure 6:**
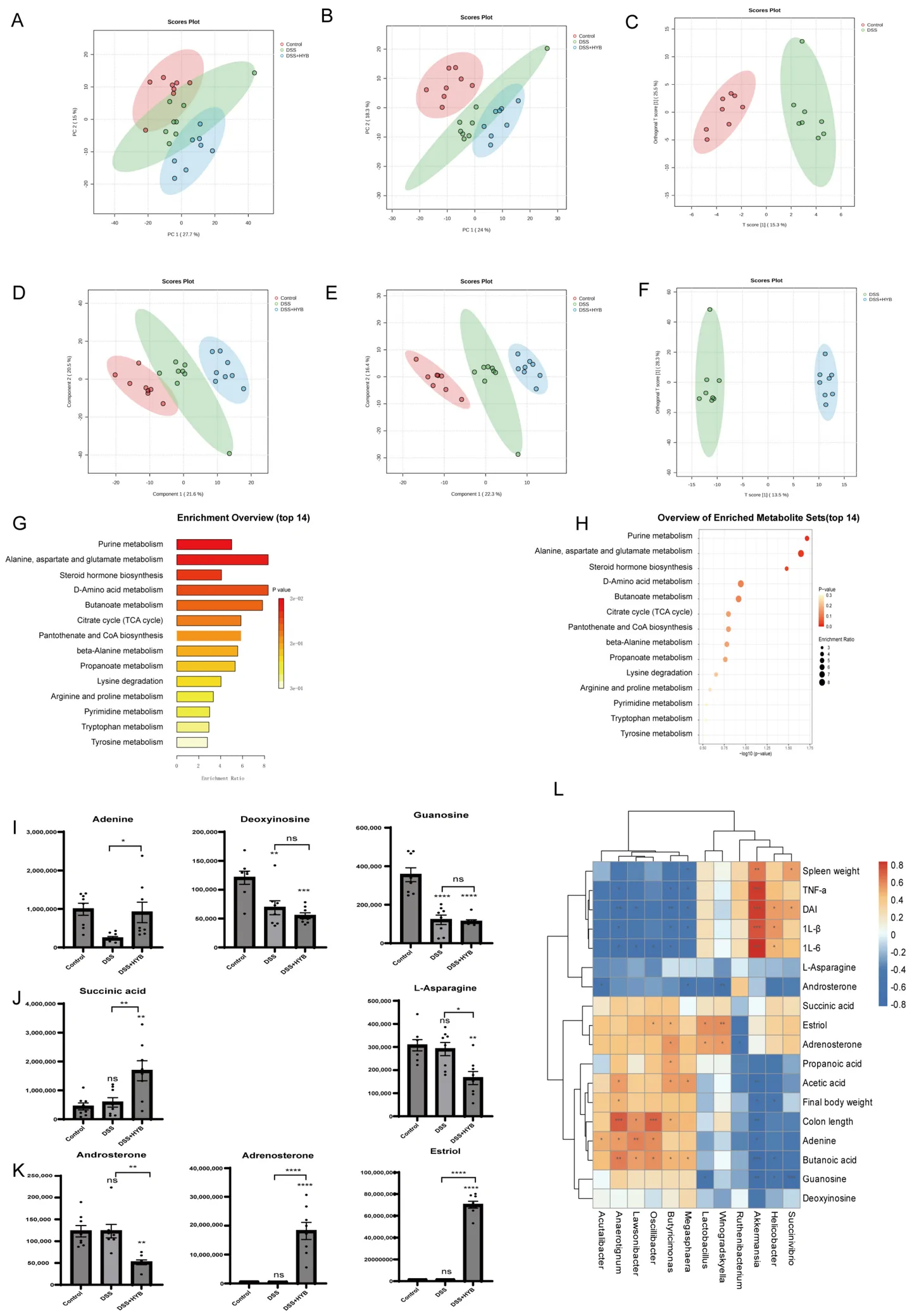
Untargeted fecal metabolomics profiling via HPLC-QTOF-MS/MS. (**A**,**B**) PCA and (**C**,**D**) PLS-DA score plots of fecal metabolites in the positive (ESI+) and negative (ESI−) modes among the three groups. (**E**,**F**) OPLS-DA score plots showing distinct metabolic clustering between (**E**) the Control and DSS groups (R^2^Y = 0.952, Q^2^ = 0.813) and (**F**) the DSS and DSS + HYB groups (R^2^Y = 0.992, Q^2^ = 0.733). (**G**) Overview of the top 14 enriched metabolic pathways. (**H**) Metabolite set enrichment analysis (MSEA). Relative abundances of key differential metabolites enriched in (**I**) purine metabolism, (**J**) alanine, aspartate, and glutamate metabolism, and (**K**) steroid hormone biosynthesis. (**L**) Spearman’s correlation heatmaps illustrate the relationships between altered fecal metabolites and colitis-associated symptoms, as well as key gut microbiota at the genus level. Data are presented as the mean ± SEM (*n* = 8 per group). * *p* < 0.05, ** *p* < 0.01, *** *p* < 0.001, and **** *p* < 0.0001 compared with the DSS group. ns indicates not significant.

Multivariate analyses, including PCA, PLS-DA, and OPLS-DA score plots, revealed clear spatial separations among the Control, DSS, and DSS + HYB groups, confirming that YB comprehensively reshaped the colitic metabolic profile (Figure 6A–F). Subsequent pathway enrichment analysis highlighted that the differential metabolites were predominantly enriched in three core hubs: purine metabolism (exhibiting the highest enrichment ratio); alanine, aspartate, and glutamate metabolism; and steroid hormone biosynthesis (Figure 6G,H). Key differential metabolites driving these shifts included adenine, deoxyinosine, and guanosine (purine metabolism); succinic acid and L-asparagine (amino acid metabolism); and androsterone, adrenosterone, and estriol (steroid hormone biosynthesis). Compared to the DSS group, YB intervention significantly upregulated the levels of adenine, succinic acid, adrenosterone, and estriol, while downregulating L-asparagine and androsterone (Figure 6I–K).

To map the microbiota–metabolite–inflammation crosstalk, we conducted Spearman’s correlation analysis (Figure 6L). The DSS-enriched *Akkermansia* positively correlated with pro-inflammatory cytokines but negatively with SCFAs and adenine. Conversely, the YB-enriched Butyricimonas and Lawsonibacter negatively correlated with inflammation and positively with SCFA synthesis. Furthermore, Lawsonibacter strongly correlated with key restored metabolites, including adenine, estriol, and adrenosterone. Collectively, these multi-omics data suggest that YB may mitigate colitis through the suppression of *Akkermansia* overgrowth, enrichment of SCFA-producing probiotics, and remodeling of host purine and steroid metabolism.

## 4. Discussion

Although Yakuchinone B (YB), a well-recognized bioactive diarylheptanoid isolated from Alpinia oxyphylla, has been reported to possess diverse biological activities, its in vivo effect on intestinal inflammation remains poorly understood. In the present study, we provide compelling evidence that YB effectively alleviates dextran sulfate sodium (DSS)-induced colitis in mice. This protective efficacy was phenotypically characterized by a marked attenuation of body weight loss, colonic shortening, and splenomegaly, alongside significant reductions in pro-inflammatory cytokine production and histopathological mucosal damage. But its long-term safety, pharmacokinetics, optimal dosage, and translational feasibility in both sexes still require further investigation, as the present study was conducted only in male mice.

Mechanistically, our findings reveal that YB supplementation rescues gut microbial diversity and profoundly reshapes the taxonomic composition of the microbiome. Specifically, YB intervention enriched beneficial commensals such as *Alistipes* and *Duncaniella*, while effectively curbing the overgrowth of *Akkermansia*. Although *Akkermansia* is often regarded as a beneficial mucin-degrading genus, the role of *Akkermansia* in colitis is context-dependent. Its enrichment in DSS-treated mice may reflect inflammation-associated mucus layer disruption and excessive mucin utilization, whereas the reduction in *Akkermansia* after YB treatment may indicate partial restoration of the mucosal microenvironment.

Beyond microbial restructuring, YB also altered the fecal metabolic profile. We observed significant modulations in key metabolic hubs, notably purine metabolism, alanine, aspartate, and glutamate metabolism, and steroid hormone biosynthesis. These altered pathways are closely related to intestinal inflammation and host–microbiota metabolic homeostasis. Purine metabolism may participate in colitis through nucleotide turnover, epithelial repair, and purinergic immune signaling. Alanine, aspartate, and glutamate metabolism reflect inflammation-associated remodeling of amino acid and energy metabolism, whereas steroid hormone biosynthesis may be linked to the immunoendocrine regulation of intestinal inflammation. Thus, these changes suggest that YB may promote broader metabolic reprogramming during the alleviation of colitis, although their causal contribution requires further validation.

In addition, YB restored the levels of major short-chain fatty acids (SCFAs), which are essential for maintaining intestinal barrier integrity and regulating local immune responses. Integrative correlation analysis further revealed close associations among gut microbial genera, altered metabolites, SCFAs, and inflammatory markers. Specifically, *Akkermansia* was positively associated with inflammatory cytokines and negatively associated with SCFAs, whereas *Butyricimonas* and *Lawsonibacter* showed opposite correlation patterns. These findings suggest that YB may alleviate DSS-induced colitis through coordinated modulation of the gut microbiota–metabolite axis. However, the causal relationships among specific microbial taxa, metabolic pathways, SCFA production, and inflammatory suppression remain to be clarified in future studies.

Nevertheless, our study demonstrated that dietary supplementation with YB robustly protects against DSS-induced colonic injury and inflammation. This insight highlights the promising potential of YB as a novel, natural, and functional dietary intervention for the prevention and management of IBD.

## 5. Conclusions

This study demonstrates that dietary supplementation with Yakuchinone B (YB) robustly protects against DSS-induced colonic injury and inflammation in mice. This protective effect is associated with a reshaping of the gut microbiota, characterized by enriched beneficial commensals *Alistipes* and *Duncaniella* and a context-dependent reduction in *Akkermansia*, along with modulations of purine metabolism, alanine/aspartate/glutamate metabolism, and steroid hormone biosynthesis and restoration of short-chain fatty acid levels. Integrative correlation analysis further reveals close associations between specific microbial genera, metabolites, and inflammatory markers, suggesting that YB alleviates colitis through coordinated modulation of the gut microbiota–metabolite axis. These findings highlight the promising potential of YB as a novel, natural, and functional dietary intervention for the prevention and management of IBD, although its long-term safety, pharmacokinetics, optimal dosage, efficacy in both sexes, and the causal mechanisms remain to be investigated.

## Data Availability

The original contributions presented in this study are included in the article. Further inquiries can be directed to the corresponding author.
